# Spatial variations in estimated chronic exposure to traffic-related air pollution in working populations: A simulation

**DOI:** 10.1186/1476-072X-7-39

**Published:** 2008-07-18

**Authors:** Eleanor M Setton, C Peter Keller, Denise Cloutier-Fisher, Perry W Hystad

**Affiliations:** 1 Geography Department, University of Victoria, PO Box 3050, STN CSC, Victoria, B.C., V8P 3W5, Canada; 2 Department of Health Care and Epidemiology, University of British Columbia, 5804 Fairview Ave., Vancouver, B.C., V6T 1Z3, Canada

## Abstract

**Background:**

Chronic exposure to traffic-related air pollution is associated with a variety of health impacts in adults and recent studies show that exposure varies spatially, with some residents in a community more exposed than others. A spatial exposure simulation model (SESM) which incorporates six microenvironments (*home indoor*, *work indoor*, *other indoor*, *outdoor*, *in-vehicle to work *and *in-vehicle other*) is described and used to explore spatial variability in estimates of exposure to traffic-related nitrogen dioxide (not including indoor sources) for working people. The study models spatial variability in estimated exposure aggregated at the census tracts level for 382 census tracts in the Greater Vancouver Regional District of British Columbia, Canada. Summary statistics relating to the distributions of the estimated exposures are compared visually through mapping. Observed variations are explored through analyses of model inputs.

**Results:**

Two sources of spatial variability in exposure to traffic-related nitrogen dioxide were identified. Median estimates of total exposure ranged from 8 μg/m^3 ^to 35 μg/m^3 ^of annual average hourly NO_2 _for workers in different census tracts in the study area. Exposure estimates are highest where ambient pollution levels are highest. This reflects the regional gradient of pollution in the study area and the relatively high percentage of time spent at home locations. However, for workers within the same census tract, variations were observed in the partial exposure estimates associated with time spent outside the residential census tract. Simulation modeling shows that some workers may have exposures 1.3 times higher than other workers residing in the same census tract because of time spent away from the residential census tract, and that time spent in work census tracts contributes most to the differences in exposure. Exposure estimates associated with the activity of commuting by vehicle to work were negligible, based on the relatively short amount of time spent in this microenvironment compared to other locations. We recognize that this may not be the case for pollutants other than NO_2. _These results represent the first time spatially disaggregated variations in exposure to traffic-related air pollution within a community have been estimated and reported.

**Conclusion:**

The results suggest that while time spent in the *home indoor *microenvironment contributes most to between-census tract variation in estimates of annual average exposures to traffic-related NO_2_, time spent in the *work indoor *microenvironment contributes most to within-census tract variation, and time spent in transit by vehicle makes a negligible contribution. The SESM has potential as a policy evaluation tool, given input data that reflect changes in pollution levels or work flow patterns due to traffic demand management and land use development policy.

## Background

Chronic exposure to traffic-related air pollution is associated with a variety of health impacts in adults, and recent studies show that some residents in a community may be more exposed than others. For example, living in proximity to busy roads has been associated with pre-term birth [1], higher cardiopulmonary mortality [2], earlier mortality [3], and higher prevalence rates of respiratory symptoms (persistent cough, asthma, wheeze, and breathlessness) [4]. Other sources of variation in exposure may also exist due to systematically higher pollution levels in some types of locations. Monitoring studies of NO_2 _provide evidence that consistently higher levels can be observed in urban areas and in transit-related environments. For example, short-term monitoring of NO_2 _in Helsinki showed that personal exposures for those participants who lived downtown were 23 percent higher than for those for participants living in suburban areas, a difference of approximately 7 μg/m^3 ^[5]. Measured levels of NO_2 _in Hong Kong suggest that short-term concentrations are as much as 17 times higher in transit environments (bus, truck, van or car) than ambient outdoor levels [6], and a study conducted in North Carolina focusing on cars specifically found levels of NO_2 _inside cars, averaged over the course of several hours driving, to be about 1.4 times higher compared to levels measured concurrently at an ambient monitoring site [7]. These studies provide evidence that exposure to traffic-related air pollution can affect population health, and that within-community spatial variations exist in exposure. Understanding where, and by how much, people may be exposed to traffic-related air pollution is important in determining the best approaches for reducing exposures and ultimately understanding health outcomes.

The objective of this study is to investigate the spatial pattern and relative magnitude of variation in chronic (annual average hourly) exposure to nitrogen dioxide (NO_2_) for workers in the Greater Vancouver Regional District (GVRD) of British Columbia. As of 2004, the GVRD had a population of approximately 2.1 million people [8]. On average, 57 percent of commuters travel outside of their home municipality to work, although this percent is lower near the downtown core and higher in the near suburbs [9]. For this study, exposure is considered to be due to inhalation only and equivalent to the level of pollution in the air near a person, not the actual amount inhaled or absorbed in the body. For the purpose of this study indoor sources of pollution are not considered, only outdoor levels associated primarily with traffic. This research marks the first time such spatially disaggregated estimates of within-community variability in exposure have been produced via simulation modelling and subsequently mapped. It also is the first example of incorporating geographic details into air pollution simulation modelling through the use of a geographic information system and data sets with high spatial resolution.

## Methods

There are two generally accepted methods for investigating characteristics (or determinants) of exposure. The first is the direct method in which personal monitoring is conducted and often linked to a time-activity diary, as for example in Georgoulis et al (2002) or DeBruin et al (2004) [10,11]. The second is the indirect method, in which pollutant concentrations in various locations (called microenvironments) are monitored in conjunction with a time-activity diary that records the amount of time a person spends in each microenvironment, as for example in Chau et al (2002) [6]. In the first case, personal monitoring studies provide the best information, but are necessarily limited to populations small enough to feasibly monitor. This is also true of the latter case; however, a simulation approach has been employed to model large populations, on the order of an entire urban area [12-15]. The simulation consists of randomly selecting from representative distributions of pollution concentration in a set of microenvironments and from sets of time-activity patterns that represent the population of interest.

It is important to note that exposure studies do not incorporate data on health outcomes, such as would occur in an epidemiological study where statistical analyses are used to assess the association between individual health outcomes and individual exposure measures. The results of exposure studies provide important information about the typical range of exposures for a certain population, as well as where and by how much the study population is exposed to potentially harmful pollutants. Information of this kind can be used to guide policy and practice aimed at reducing exposures and thereby potentially improving the health of a population.

For this research, a random selection indirect approach is used to simulate exposures, based on the following six microenvironment (ME) equation:

(1) ***E ***= [(*C*_*h *_× *t*_*h*_) + (*C*_*w *_× *t*_*w*_) + (*C*_*oi *_× *t*_*oi*_) + (*C*_*o *_× *t*_*o*_) + (*C*_*vw *_× *t*_*vw*_) + (*C*_*vo *_× *t*_*vo*_)]/*T*

***E ***is the total exposure expressed in pollution concentration units (μg/m^3^); *C*_*h *_is the pollutant concentration at *home indoor*; *C*_*w *_is the pollutant concentration at *work indoor*; *C*_*oi *_is the pollutant concentration at *other indoor*; *C*_*o *_is the pollutant concentration *outdoor*; *C*_*vw *_is the pollutant concentration for *in-vehicle to work*; *C*_*vo *_is the pollutant concentration for *in-vehicle other*; *t*_h_, *t*_w_, *t*_oi_, *t*_o_, *t*_vw _and *t*_vo _are the time spent in each respective microenvironment, based on the time-activity pattern; and *T *equals the duration of time activity pattern. For this study, ***E ***is referred to as the total exposure, and the value associated with each microenvironment variable as the partial exposure. As per Equation 1, there will be six partial exposure estimates, one for each ME listed on the right hand side of the equation. For this research, ***E ***refers to exposure for a group, i.e., workers, rather than to individuals. Exposures are simulated through iterative random sampling of representative distributions of the terms in Eq (1).

This approach is based on work by Duan, Klepeis and Ott [16-18] and has been used extensively by the US Environmental Protection Agency (US EPA) in support of setting air quality management objectives and standards [12,14,19-21]. Many of the exposure simulations conducted using this approach have been applied to estimate a single distribution of exposure for an entire urban population and therefore incorporate demographics to represent the population[12,13,22-27]. The objective here, however, is to investigate variability in exposure due to relative location within a metropolitan area and its suburbs and the spatial pattern of pollution, rather than variability due to demographic patterns. For this reason, a unique spatial exposure simulation model (SESM) was developed using a geographic information system (ESRI ArcGIS 9.1^©^) and custom C^++ ^programming.

### Data

**Figure 1:**
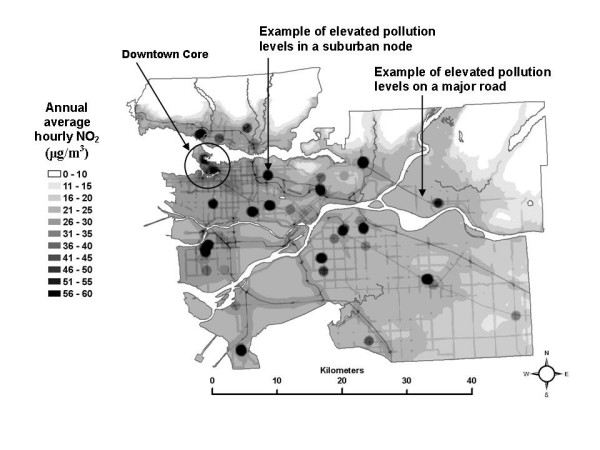
Spatial estimate of annual average NO_2 _levels in the Greater Vancouver Regional District study area.

Five key datasets were employed in the SESM:

1) Time-activity data from the Canadian Human Activity Pattern Survey [28], conducted in 1994, were used as a basis for simulation in the SESM. Time reported as spent in a variety of locations over 24 hours was aggregated for each of 756 adult time-activity patterns into the six MEs used for this research: *home indoor*, *work indoor*, *other indoor *(including time spent shopping or in restaurants, for example), *outdoor*, *in-vehicle to work*, and *in-vehicle other *(all non-work transit time). In order to incorporate weekday/weekend and summer/winter variations in the amount of time spent in different MEs over a year, the time-activity pattern records were grouped into three categories: non-workers summer (320 records), non-workers winter (278 records), and workers (178 records), which provide the required distributions for random sampling of the amount of time spent in each ME. In the SESM, this same pool of time-activity patterns is used for the simulation in each census tract, thus controlling for variation due to demographic differences.

2) Work flow data for all people aged 15 and over reporting employment, based on a 20 percent sample in each census tract on May 15^th^, 2001, were purchased from Statistics Canada. For each census tract, the numbers of workers going from the census tract of their residence to another census tract in the study area for work were used to develop frequency-weighted work pair lists (distributions) for use in the SESM. Workers who had a place of employment within the census tract of their residence were excluded, as were workers who had a place of employment outside the GVRD study area.

3) Nitrogen dioxide was chosen for study as it is recognized to be an indicator of traffic-related air pollution [29-37]. A spatial estimate of annual average NO_2 _levels (Figure 1), developed by researchers at the University of British Columbia using the land use regression (LUR) method with field monitoring conducted in 2003, was employed and is fully described in [38]. Briefly, the method uses linear regression to relate surrounding geographic variables to field measurements of the pollutant of interest, and the resulting model is used to predict pollution levels for every cell in a grid that covers the entire study area. The grid spacing is usually very fine (i.e., 5 metres), and captures pollution gradients associated with roadways and dense urban and commercial development. The most important features of the spatial estimates are that gradients in NO_2 _with distance from roadways are captured, as are higher concentrations of NO_2 _in commercially developed areas and the regional gradient from the more polluted urban core, to the less polluted rural areas. This level of detail in the spatial allocation of NO_2 _allows the SESM, as employed in this study, to differentiate among census tracts based on their location in the study area and among *in-vehicle*, *work indoor*, and *home indoor *MEs.

4) Spatial property assessment data were used to identify the geographic location of every property in the study area for which taxes are assessed as well as the primary use of the building on the property. Within each census tract, property locations (either residential or commercial) were used to constrain the SESM to areas where these buildings exist, rather than assuming a homogeneous spatial distribution. In addition, since outdoor air pollution does not always fully infiltrate into indoor areas, indoor/outdoor (I/O) ratios are applied according to building type based on a review of available indoor/outdoor monitoring studies, for example, [39-47]. Residential buildings were assigned an I/O ratio of 0.70, large office buildings 0.35, manufacturing, industrial, and civic buildings 0.50, and small stores, services, and restaurants 0.70.

5) A digital road dataset was acquired for the study area from DMTI Spatial Inc. under an academic research agreement. This dataset was converted into a GIS network using ArcGIS 9.1 ^©^, thereby allowing for the identification of the shortest route, assuming travel by car, between two points while observing appropriate travel restrictions such as one way streets and speed limits. For every home census tract, the shortest route based on time for every work census tract listed in the work pair file was identified and saved as a unique GIS file using a specific identifier (i.e., originCT10_destinationCT_205) with attributes including total distance and total time associated with the route. In total, 34,782 GIS files were created, representing the shortest routes between each home census tract and all associated work census tracts.

### Developing distributions of NO_2 _concentrations

Given the spatial pollution estimate, routes between home and work census tracts, building locations and associated I/O ratios, a geographic information system was used to develop distributions of NO_2 _for each ME that are unique to each census tract in the study area. For a specific census tract, the distribution of NO_2 _levels for the *home indoor *ME is comprised of values extracted from the NO_2 _surface for each residential building location (assumed to be the centroid of each property parcel) in the census tract and multiplied by the I/O ratio of 0.70. For the *work indoor *ME, the pollution distribution is developed the same way, extracting NO_2 _levels at commercial building locations within the work census tract and multiplying by I/O ratios of 0.35, 0.50, or 0.70, according to building type. Pollution distributions for the *other indoor *ME are based on sampling the pollution surface at commercial locations within 5 kilometers of the residential census tract and again multiplying by the appropriate I/O ratio. Similarly, for the *outdoor *ME, a regularly spaced grid of sample points is used to extract NO_2 _values from the surface within 5 kilometers of the residential census tract. For the *in-vehicle other *ME, the road network is used to create sample points for extracting NO_2 _values along roadways within 5 kilometers of the residential census tract; these values are then averaged to provide a single *in-vehicle other *value for the census tract. A distance of 5 kilometers was chosen to represent the average distance people travel from home on non-work related trips based on a review of studies reporting a typical range between 2 and 8 kilometers [48-50]. As well, the SESM results were found to be insensitive to variations in the distance chosen between 2.5 and 7.5 kilometers. For the *in-vehicle to work *ME, distributions are made up of the length-weighted average pollution level along the shortest route between each home-work pair associated with the census tract. No I/O ratios are applied to the *outdoor*, *in-vehicle othe*r or *in-vehicle to work *MEs. It is important to note here than no indoor sources of NO_2 _are included in the simulation. For indoor microenvironments, the distributions for NO_2 _levels represent the amount of NO_2 _generated by traffic that infiltrates indoors.

### Random sampling algorithm

Once the pollution concentration distributions for each census tract are developed, random sampling of these and other key variables is used to calculate total exposures for a year. Certain assumptions were made based on the need to use daily time activity patterns to simulate annual exposures, most importantly that workers have the same time-activity pattern on all workdays in the year, and have a random non-work pattern on each weekend day of the year. Beginning with the first census tract, one commuter time-activity pattern is randomly selected to represent all workdays in a year, a work destination is randomly chosen from the frequency-weighted distribution of possible work pairs for that census tract, and pollution concentrations are randomly chosen from the appropriate distributions for the MEs. Total exposure is then calculated as a time-weighted average of these randomly selected pollution levels for each ME, as per Equation (1), as are the partial exposures associated with each ME. For summer weekends (53 days between March 21^st ^and September 21st), a non-commuter summer time-activity pattern is randomly selected, the appropriate distributions sampled for pollution levels, and exposure calculated as per Equation (1). This is repeated 53 times, and the average taken to represent the total and partial exposures for summer weekends. A similar procedure is used to calculate the total and partial exposures for winter weekends (53 days between September 22^nd ^and March 20^th^). Results for workdays and summer and winter weekends are then combined to provide a time-weighted, seasonally adjusted estimate of total exposure to annual average hourly NO_2_, as per Equation (2):

(2) ***E*_*y *_**= [{(W_a _* 0.72) + (W_ws _* 0.28)}* 0.50] + [{(W_a _* 0.72) + (W_ww _* 0.28)} * 0.50]

Where ***E*_*y *_**is a single calculation of the annual total exposure; W_a _is the exposure during weekdays; W_ws _is the exposure during summer weekends; W_ww _is the exposure during winter weekends; the weight 0.72 represents the proportion of working days in summer or winter; the weight 0.28 represents the proportion of weekend days in summer or winter; and the weight 0.50 gives the summer and winter components equal weight in the sum.

This set of calculations is repeated 10,000 times for a census tract, producing distributions of total exposure estimates as well as partial exposure estimates for each ME, with 95% confidence of estimating exposure at any percentile to within +/-1 percentile [51]. This procedure is then repeated for each census tract in the study area, and the resulting distributions can be compared statistically as well as visually through mapping. For example, the median (referred to as the 50^th ^percentile values) of the total or partial exposure distributions in each census tract can be mapped to identify spatial patterns. The 10^th ^percentile and 90^th ^percentile values can be similarly mapped and compared to identify the lower and upper ranges of the exposure distributions. The differences between the 10^th ^and 90^th ^percentiles can be calculated and mapped to show where variability within census tracts is highest, i.e., where the range between the 10^th ^and 90^th ^percentile values is largest.

### Model limitations

Due to the specification of the SESM as applied here, results must be interpreted with caution, under the following caveats. (1) Results apply to those commuting by vehicle, not working in the census tract of residence, and who have a fixed place of employment all year. Therefore, the SESM does not reflect exposures of people who work in transit-related occupations (e.g., bus, taxi, truck drivers), who regularly work outdoors, who have a variable work location (e.g., real estate sales, home care workers), or who use other modes of transportation. (2) Results do not reflect real measured personal exposures, but are estimates of the distribution of exposure within census tracts for a group of people (i.e. workers). (3) The results reported here are specific to NO_2 _and may reflect exposure to other traffic-related air pollutants. The results do not include any indoor sources of NO_2 _such as gas stoves or work-related equipment. (4) SESM results are sensitive to the use of different methods for estimating pollution levels, e.g., spatial interpolation based on fixed-site monitoring or LUR. Here, the most spatially detailed estimate of NO_2 _levels available for the study area was employed. Less detailed estimates, such as might be produced via spatial interpolation from fixed-site air quality monitors, may produce results that differ from those reported here.

## Results

**Figure 2:**
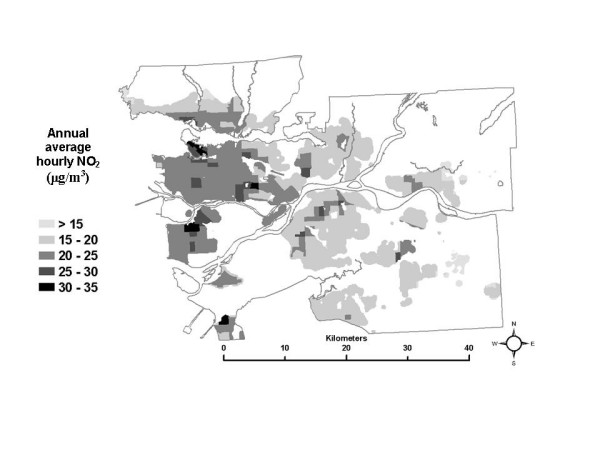
Spatial distribution of the median total exposure.

Looking first at the total exposure estimates, the SESM distributions show that, on average, total exposures range from as high as 35 μg/m^3 ^of annual average hourly NO_2 _in the downtown core to as low as 8 μg/m^3 ^in rural areas. When the 50^th ^percentile values for the distributions in each census tract are mapped (Figure 2), this generally decreasing gradient based on distance from the urban core is apparent, as are hot spots of elevated total exposures in suburban areas where nodes of dense commercial development exist (See Figure 1 for elevated levels of NO_2 _associated with urban core, suburban developments and roadways). Note that the maps presented do not show any data for areas where there is less than one residential building per 500 metres, in order to avoid giving visual importance to unpopulated regions of large census tracts.

Comparing the map of the 50^th ^percentile of the total exposure estimates (Figure 2) to the map of the spatial estimate of NO_2 _used in the analysis (Figure 1), it appears that median total exposures generally follow the same spatial pattern as the pollution estimate. This pattern remains the same at the 10^th ^and 90^th ^percentile levels of the total exposure distributions (not shown here), although the 10^th ^percentile levels are systematically lower and the 90^th ^percentile levels are systematically higher than the 50^th ^percentile levels shown in Figure 2. These results are not unexpected, since it would not be unreasonable to predict higher exposures where pollution is higher, based on the estimated ambient pollution surface. It also should be noted that the magnitude of the total exposure estimates is generally lower than the ambient pollution levels due to the use of indoor/outdoor ratios in the SESM to adjust for the lower levels of ambient pollution infiltrating inside residences and places of work.

**Figure 3:**
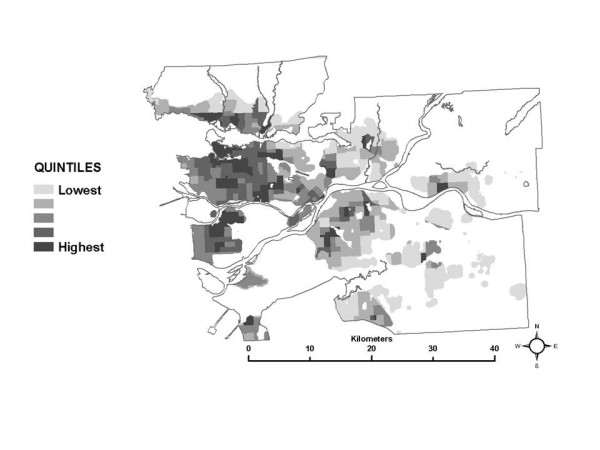
Spatial distribution of the median partial exposure associated with the *home indoor *microenvironment.

Next, looking at the partial exposure estimates associated with each ME, unique spatial patterns are also evident, as shown using quintiles of the median of the exposure distributions in each census tract in Figures 3 through 8. The spatial pattern of the median partial exposure associated with the *home indoor *ME, shown in Figure 3, closely resembles the pollution estimate (Figure 1), with high median partial exposures corresponding to high pollution areas. This makes intuitive sense since this is the ME in which the most hours per day are spent, so pollution levels at residences have a substantial influence on total exposure.

**Figure 4:**
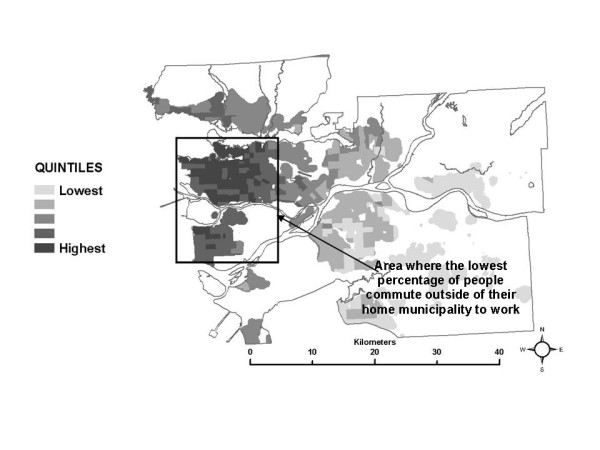
Spatial distribution of the median partial exposure associated with the *work indoor *microenvironment.

**Figure 5:**
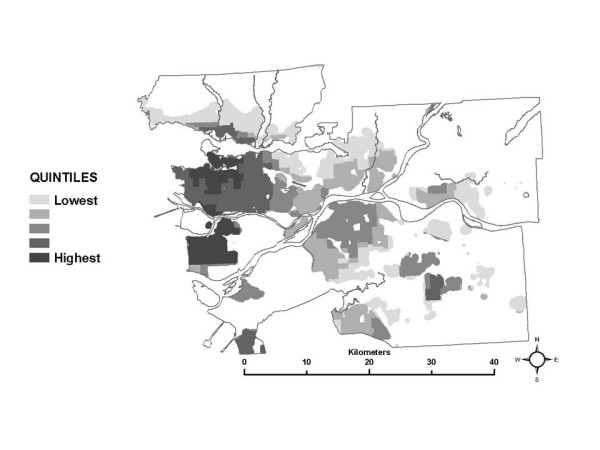
Spatial distribution of the median partial exposure associated with the *other indoor *microenvironment.

**Figure 6:**
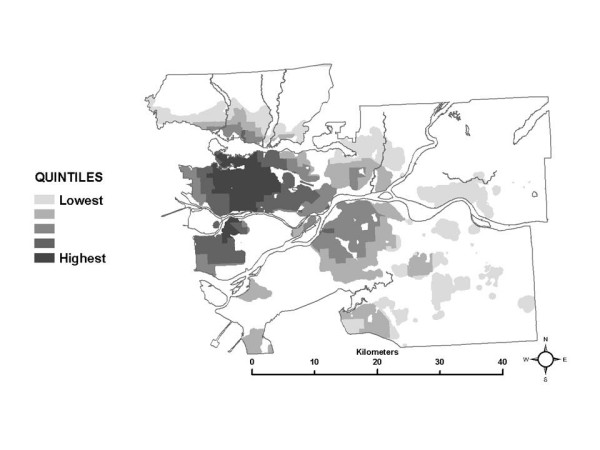
Spatial distribution of the median partial exposure associated with the *outdoor *microenvironment.

**Figure 7:**
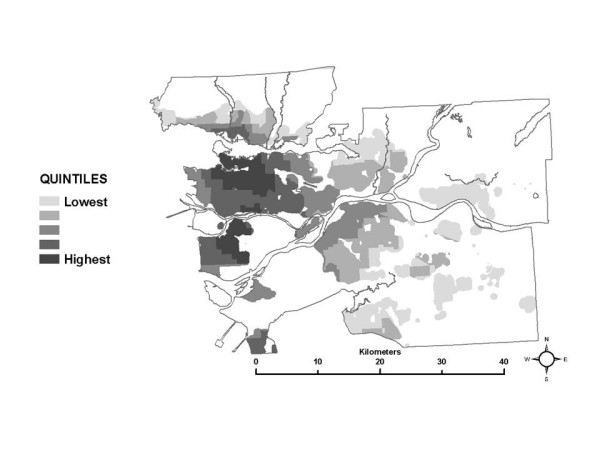
Spatial distribution of the median partial exposure associated with the *in-vehicle other *microenvironment.

**Figure 8:**
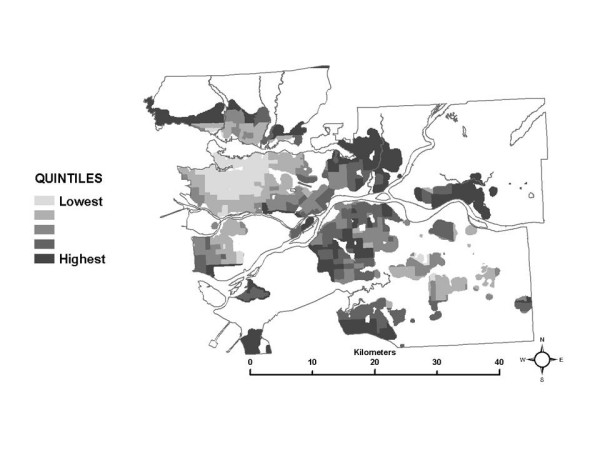
Spatial distribution of the median partial exposure associated with the *in-vehicle to work *microenvironment.

Figure 4 shows quintiles of the median partial exposure associated with the *work indoor *ME. In this case, median partial exposure is highest in the urban core and surrounding densely developed areas. In the SESM, partial exposure associated with the *work indoor *ME is based on NO_2 _levels in the census tracts where residents report working, according to the Statistics Canada work flow data. Higher median exposure estimates in a census tract therefore suggest that most of the workers living there are working in other census tracts where pollution levels are similar to each other and relatively high. It seems reasonable to suggest that the pattern of higher median partial exposures for workers living in and around the urban core associated with the *work indoor *ME is due to a preponderance of work locations within the same general area, indicating relatively short commute distances to work destinations within the more highly polluted urban core and nearby areas. This interpretation is supported by local planning documents, that report that the municipalities within the area indicated in Figure 4 have the lowest percentages of people who go to other municipalities to work [9].

Quintiles of the median partial exposure associated with the *other indoor*, *outdoor*, and *in-vehicle other *MEs are shown in Figures 5, 6 and 7. In the SESM, these partial exposures are based on pollution levels at commercial locations, outdoors, and along roads within 5 km of each individual census tract, and so reflect the larger scale regional variation in NO_2 _levels: higher in the urban core and nearby developed areas and decreasing toward rural areas.

The spatial pattern in the median of the partial exposure distributions associated with the *in-vehicle to work *ME is shown in Figure 8. Here, census tracts with the highest quintile of median partial exposure are located in suburban and rural areas. For this ME, partial exposures in a particular census tract are based on the length-weighted average NO_2 _level along the shortest routes between that census tract and all other census tracts where people report working. In addition, the frequency with which each work census tract is reported as a destination is incorporated in the SESM calculations, so the distribution of partial exposures is most influenced by the NO_2 _levels along the routes traveled to the most-often reported work census tracts. The spatial pattern seen here is consistent with longer commutes from suburban and rural areas on highways and major roads where NO_2 _levels are elevated.

**Figure 9:**
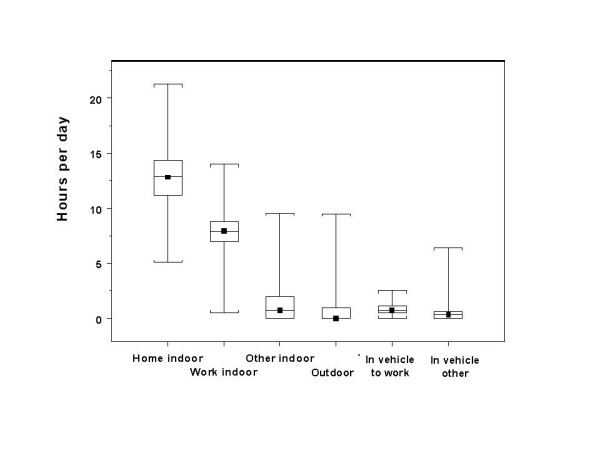
Distribution of hours per day spent in each microenvironment.

While the medians of the partial exposures associated with the six MEs included in the SESM have distinct patterns, each partial exposure has a different level of influence on the pattern of total exposures. Box plots of the amount of time spent in each ME for all of the time-activity patterns used in the SESM are shown in Figure 9. Each box plot shows the minimum and maximum (the lowest and highest horizontal line), and the 25^th ^percentile, median and 75^th ^percentile level (bottom, middle, and top of box) of time spent in the associated ME. Within a 24 hour period, the amount of time spent in the *home indoor *ME is the highest, with a median of about 13 hours, followed by the *work indoor *ME, with a median of about 8 hours. Median time spent in each remaining ME is less than one hour each. The SESM produces a time-weighted, seasonally adjusted average of total exposure based on the input set of 24 hour time-activity patterns, so even though pollution levels may be high in some MEs (i.e., *in-vehicle to work*), the relatively small amount of time spent in these MEs in any 24 hour period reduces the importance of these exposures in terms of the annual average hourly total exposure.

The previous results have focused on the 50^th ^percentile levels of the distributions for total and partial exposures in the study area, and regional gradients in exposure are evident; however, additional detail can be found by comparing other points of the distributions produced for each census tract. Table 1 provides a summary of the mean, median, and standard deviations at the 10^th^, 50^th^, and 90^th ^percentiles for the total and partial exposure distributions produced for each of the 382 census tracts in the study area. Also reported is the range between the mean of the 10^th ^and 90^th ^percentiles for all census tracts. Looking down the columns in Table 1 for the 10^th^, 50^th^, and 90^th ^percentiles, the relative contribution of each ME to the total exposure is apparent, and is associated with the time spent in each ME, as discussed earlier. Of particular interest to note is the relatively negligible partial exposure associated with the *in-vehicle to work *ME. Also of interest are the ranges associated with each ME, shown in the last column of Table 1. These are produced by subtracting the mean of the 10^th ^percentile values for all census tracts from the mean of the 90^th ^percentile values for all census tracts, and indicate the variability of exposure within census tracts as opposed to between census tracts. The mean range for total exposure is approximately 5 μg/m^3^, while the mean ranges associated with each ME are 1 μg/m^3 ^or less, with the exception of the *work indoor *ME, shown in bold. The mean range for the *work indoor *ME is almost 7 μg/m^3^, which is far higher than that of any other ME. More detailed discussion of the very low partial exposure estimates associated with the *in-vehicle to work *ME, and the very high range in partial exposure estimates associated with the *work indoor *ME is provided next.

**Table 1:** Descriptive statistics of the exposure estimate distributions in the 382 census tracts in the Greater Vancouver Regional District study area

**Exposure estimates**		**Annual average hourly NO_2 _(μg/m^3^)**			
	
**Descriptive statistics**		**10^th ^** **percentile** ** (n = 382)**	**50^th ^** **percentile** ** (n = 382)**	**90^th ^** **percentile** ** (n = 382)**	**Range ** **(10^th ^– 90th)**
Total exposure	Mean	18.87	20.96	23.89	5.02
	Median	18.61	20.62	23.58	
	st. deviation	3.24	3.48	3.49	
					
Home indoor	Mean	14.88	15.39	15.89	1.01
	Median	14.48	15.00	15.47	
	st. deviation	3.50	3.63	3.76	
					
Work indoor	Mean	3.79	6.40	10.60	**6.81**
	Median	3.72	6.39	10.52	
	st. deviation	0.37	0.64	0.76	
					
Other indoor	Mean	1.76	2.09	2.44	0.68
	Median	1.67	1.99	2.35	
	st. deviation	0.43	0.50	0.58	
					
Outdoor	Mean	2.36	2.78	3.24	0.88
	Median	2.39	2.82	3.28	
	st. deviation	0.36	0.42	0.49	
					
In-vehicle to work	Mean	0.19	0.54	1.06	0.87
	Median	0.16	0.52	0.99	
	st. deviation	0.15	0.24	0.36	
					
In-vehicle other	Mean	1.32	1.53	1.76	0.44
	Median	1.33	1.54	1.77	
	st. deviation	0.18	0.21	0.25	

Although the amount of time spent in the *in-vehicle to work *ME was known to be low, it was expected that the use of a spatial estimate of NO_2 _that captured elevated concentrations along roadways and the use of GIS to identify routes along those roadways would act to give additional importance to the contribution of this ME to total exposure. This clearly was not the case, and may be due to some routes (or portions of routes) following local roads where levels are not particularly elevated in the pollution estimate. The summary of LUR NO_2 _levels associated with residential locations and different road classes in the study area, provided in Table 2, supports this interpretation. The mean annual average hourly NO_2 _level at each of the residential locations in the study area is 28 μg/m^3^, compared to 34 μg/m^3 ^on highways, freeways and arterial roads, and 26 μg/m^3 ^or less on all other classes of roads. Also notable is the relatively low percentage of highways, freeways and arterial roads (17%) in the study area compared to other road classes with lower NO_2 _levels. Therefore, only long commutes on major roads will have relatively high partial exposures, and are note frequent enough in the study area to have a significant influence on total exposure. Another contributing factor could be the use of shortest paths in the model, solved to optimize total time based on speed limits, thus representing the best case scenario where there is no congestion or stop and go traffic and so may underestimate actual exposures.

**Table 2:** Estimated pollution levels associated with residential locations and different road classes

**Location**		**Annual average hourly NO_2 _(μg/m^3^) ** **based on the LUR estimate**			
	
	**percent**	**min**	**max**	**mean**	**sd**
Residences	100	6	56	28	8
freeway/highway/arterial/ramp	17	15	56	34	9
Collector	17	2	56	26	8
local/strata/lane	66	2	56	26	8
Rural	0.2	11	39	19	5

A more detailed look at the *work indoor *ME results provides additional information that aids in the explanation of the high range observed in the partial exposure associated with this ME. The SESM results suggest workers in the top 10 percent of the exposure distribution (90^th ^percentile) could be exposed to, on average 6.8 μg/m^3 ^more annual average NO_2 _than workers in the bottom 10 percent of exposure distribution (10^th ^percentile) within the same census tract. The mean ratio of the 90^th ^percentile to the 10^th ^percentile for all census tracts is 2.8, indicating that on average, workers in the top 10 percent could have exposures 2.8 times greater than workers in the bottom 10 percent of exposures who live in the same census tract. The effect of this variation on the total exposure is diminished by the influence of time spent in other MEs, producing a mean ratio of the 90^th ^percentile to 10^th ^percentile total exposure of 1.3.

**Figure 10:**
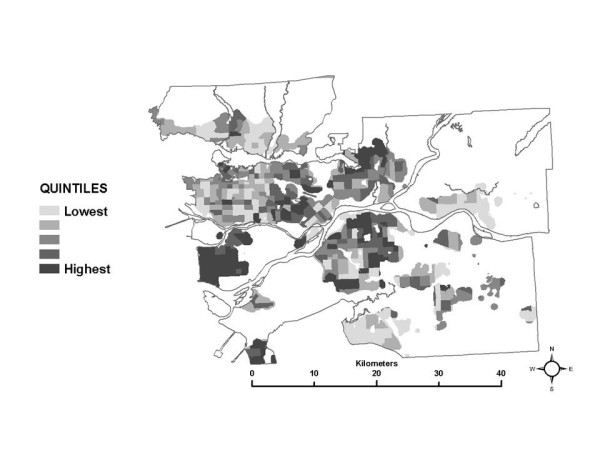
Spatial variability in partial exposure associated with the *work indoor *microenvironment (range between 10^th ^and 90^th ^percentile).

**Figure 11:**
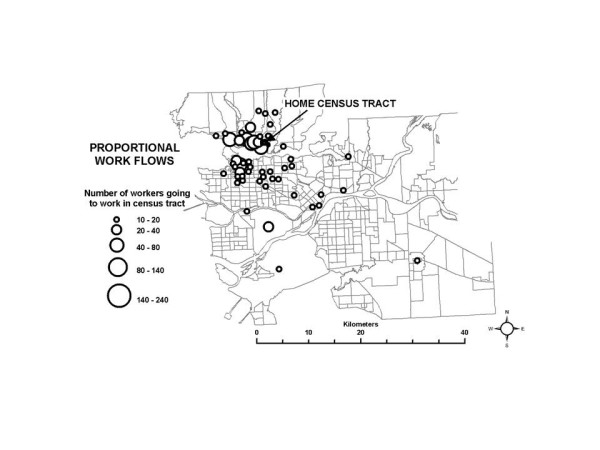
**An example of work flow patterns for selected census tracts with a low range in partial exposure associated with the *work indoor* microenvironment.** (a) Census tract near the urban core with a low range. (b) Census tract in a suburban area with a high range.

**Figure 12:**
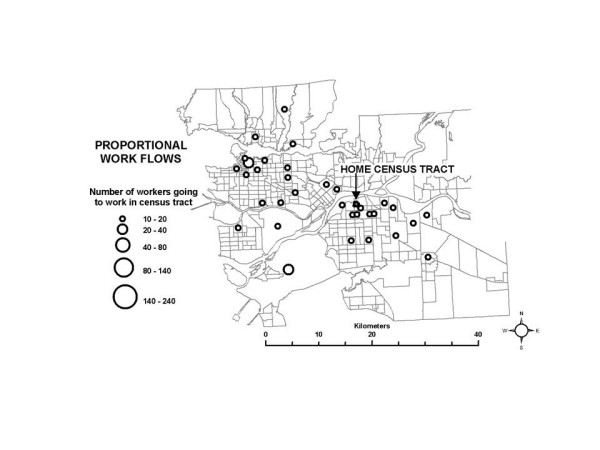
**An example of work flow patterns for selected census tracts with a high range in partial exposure associated with the work indoor microenvironment** (a) Census tract near the urban core with a low range. (b) Census tract in a suburban area with a high range.

In the SESM, distributions of partial exposure associated with the *work indoor *ME are based on the work flow frequency data specific to each census tract in conjunction with the time-activity pattern data. A large range in a census tract for the partial exposure distribution for this ME suggest that workers go to a set of work census tracts with diverse pollution levels, which is plausible for suburban and rural census tracts. Conversely, a low range suggests that most workers go to census tracts which are all relatively similar in terms of pollution levels, and this is likely true of census tracts in and near densely developed areas such as the urban core. A map of the 10^th ^– 90^th ^percentile range for the partial exposure distributions associated with the *work indoor *ME is shown in Figure 10. Larger ranges in the partial exposures associated with the *work indoor *ME are in fact more frequent in the suburban areas, and less frequent near the urban core. This interpretation is also supported by the proportional work flows shown in Figures 11 and 12, for example. Figure 11 shows that in a census tract with a low range in partial exposure, the majority of workers are going most often to census tracts relatively close to the home census tract. Figure 12 shows that in a census tract with a high range in partial exposure, workers visit a number of census tracts at varying distances from the home census tract in relatively equal proportions, where pollution levels may vary widely.

## Discussion

The simulation results suggest that workers in the GVRD may experience an annual average exposure to traffic-related NO_2 _ranging from as low as 8 μg/m^3 ^to as high as 35 μg/m^3^, with the average of the mean total exposures across all census tracts being 21 μg/m^3 ^(SD 3.5). The level of exposure is closely associated with the pollution level in the census tract of residence, which ranges from 8 μg/m^3 ^to 56 μg/m^3 ^(mean = 30 μg/m^3^, SD 7.5 μg/m^3^) annual average hourly NO_2_, based on an area weighted average of the LUR values within each census tract. These results appear to be reasonable in comparison with existing short-term personal monitoring studies conducted elsewhere (none were conducted in the study area), and are most similar to results from a study of adults from eight cities in Switzerland. In that study, the average total personal exposure to NO_2 _in a sample of adults in eight cities in Switzerland was 27 μg/m^3 ^compared to an average ambient level of 31 μg/m^3^, with approximately 7 μg/m^3 ^of personal exposure identified as being due to NO_2 _originating indoors from gas cooking and smoking [39].

Table 3 provides a summary of additional personal monitoring studies of NO_2_. While the average of the SESM results in this paper is generally within the ranges shown, direct comparisons are difficult to make due to the inclusion of indoor sources at home and at work in the personal monitoring results. An important caveat must also be included here: the SESM produces an estimate of annual average hourly exposure, whereas the personal monitoring studies report the average hourly exposure for a single day. To evaluate the SESM results, personal monitoring for representative samples of workers for each census tract would be required for a period of time that would adequately represent an entire year.

**Table 3:** Summary of NO_2 _levels measured in personal monitoring studies

**Author**	**Location**	**Number ** **of study** ** subjects**	**Total personal ** **exposure to NO** **_2 _(*u*g/m^3^)**	**Ambient ** **NO_2 _(*u*g/m^3^)**	**Study group**
Quackenboss et al (1986) [54]	Wisconsin	350	16 – 44	12 – 17	general population
Spengler et al (1994) [55]	Los Angeles basin	682	64	70	general population 50^th ^percentile
Levy (1998) [53]	International (18 cities)	14 – 117	22 – 103	24 – 105	Adults
Monn et al (1998) [39]	Switzerland	500	27	31	working adults
Rotko et al (2001) [5]	Helsinki	176	25	24	Adults
Kousa et al (2001) [52]	Basel, Prague	85	30 – 43	36 – 61	urban adults
Lai et al (2004) [41]	Oxford, UK	50	29	27	Adults
Nerriere et al (2005) [56]	France	~250	15 – 42	17 – 75	adults and children

The simulation results presented here also show that within census tract variability is due to different work flow patterns among workers in census tracts. The partial exposures at the 90^th ^percentile for *work indoor *(i.e., in the top ten percent of workers) on average can be 6.8 μg/m^3 ^higher than the partial exposure at the 10^th ^percentile (i.e., in the bottom ten percent of workers) in the same census tract, which translates into a partial exposure 2.8 times higher, and a total exposure of 1.3 times higher. The largest variability is generally found in suburban areas where workers travel to a large number of different work destinations. This is a new insight, in terms of the magnitude of variability in exposure.

Time spent commuting to work in a vehicle contributed a negligible amount to estimated exposure. This finding was somewhat unexpected; however, some empirical evidence exists that supports the conclusion that exposures encountered while in transit do not contribute significantly to total exposure when measured for a full 24 hour period or longer, or when there are other sources of the pollutant that are not associated specifically with traffic, as is the case for NO_2 _(i.e., gas stoves and heaters). In Oxford UK, a study using the EXPOLIS monitoring methodology found personal 24 hour exposures to NO_2 _(mean 24.5 μg/m^3^, SD 1.7) were similar to levels measured indoors at residences with gas stoves (mean 22.3 μg/m^3^; SD 1.8) and indoors at workplaces (mean 29.6 μg/m^3^, 1.5) [41], which could reasonably be interpreted as an indication of no other significant sources of exposure other than indoor at home and work. In Helsinki, as part of the EXPOLIS study, Rotko, Kousa et al (2001) found no association between time spent in commute (categorized as less than or more than 1 hour) and personal 24 hour NO_2 _exposure, even in the absence of gas stoves (only 9 of the 176 residences monitored used gas stoves). Similarly, a commute time greater than 1 hour was not significantly associated with NO_2 _exposure for a combined group of EXPOLIS subjects from Helsinki, Basle, and Prague [52]. Conversely, in a personal monitoring study of NO_2 _exposure conducted in 18 cities around the world, commute time exceeding one hour was found to be significantly correlated with total personal exposure [53]. In Levy (1998), the mean personal NO_2 _exposure for people with a commute longer than 1 hour was 60 μg/m^3^, compared to 56 μg/m^3 ^for people with commutes of less than 1 hour, a relatively small difference in terms of the total exposure. So, for people employed in non-transit related occupations, for which typical round-trip commutes are under 1 hour, it seems reasonable to conclude that the simulated results for partial exposure associated with the *in-vehicle to work *ME presented here would be unlikely to be significantly higher even if improvements to the SESM could be incorporated. A final note of caution is warranted here. The lack of importance of the *in-vehicle to work *ME to total exposure may be reasonable for NO_2_, but in the case of other pollutants, such as some VOCs, where there may be few indoor sources and extremely elevated levels on roadways, exposure while in a vehicle may be much more important.

## Conclusion

A spatial exposure simulation model was described and used to estimate within-community variability in exposure estimates to traffic-related annual average hourly NO_2_(μg/m^3^). The results produced show that while time spent in the *home indoor *ME contributes most to between census tract variation in exposures, time spent in the *work indoor *ME contributes most to within census tract variation, and time spent in vehicles makes a negligible contribution to annual average exposure estimates.

Although the simulation results represent exposure estimates rather than actual exposures, the explicitly spatial perspective represented here has utility for informing targeted air quality policies meant to reduce exposures and environmental inequity in terms of exposure to traffic-related air pollution. All residents of an area would benefit from an overall decrease in traffic-related air pollution, such as might be achieved by further reducing tail-pipe emissions or switching to alternative fuels. If, however, the goal of policy is to reduce exposures for those most affected, either the spatial pattern of pollution or the spatial patterns of where people live and where they work bears further scrutiny. No doubt changes in one pattern will be caused or influenced by changes in the others. The SESM has future potential as a policy evaluation tool, given input data that reflect changes in pollution levels or work flow patterns due to traffic demand management and land use development policy.

## List of Abbreviations

CHAPS: Canadian Human Activity Pattern Study; GVRD: Greater Vancouver Regional District; I/O: Indoor outdoor ratio; LUR: Land use regression; ME: Microenvironment; NO_2: _Nitrogen dioxide; SESM: Spatial exposure simulation model; US EPA: United States Environmental Protection Agency; μg/m^3: ^Micrograms per cubic metre.

## Competing interests

The authors declare that they have no competing interests.

## Authors' contributions

EMS was responsible for the conceptual design of the study, including the development and implementation of the SESM, and for preparing the draft manuscript. CPK and DCF provided feedback on study design and interpretation of results. PWH collaborated with EMS on data acquisition and processing. All authors reviewed and approved the final manuscript.
